# Pharmacogenetic genotype and phenotype frequencies in a large Danish population-based case-cohort sample

**DOI:** 10.1038/s41398-021-01417-4

**Published:** 2021-05-18

**Authors:** Carin A. T. C. Lunenburg, Janne P. Thirstrup, Jonas Bybjerg-Grauholm, Marie Bækvad-Hansen, David M. Hougaard, Merete Nordentoft, Thomas Werge, Anders D. Børglum, Ole Mors, Preben B. Mortensen, Christiane Gasse

**Affiliations:** 1 Department of Affective Disorders, Aarhus University Hospital Psychiatry, Aarhus, Denmark; 2 Department of Clinical Medicine, Aarhus University, Aarhus, Denmark; 3 Department of Biomedicine, Faculty of Health, Aarhus University, Aarhus, Denmark; 4 The Lundbeck Foundation Initiative for Integrative Psychiatric Research, iPSYCH, Aarhus/Copenhagen, Denmark; 5 Center for Genomics and Personalized Medicine, Aarhus University, Aarhus, Denmark; 6 Danish Center for Neonatal Screening, Statens Serum Institut, Copenhagen, Denmark; 7 Mental Health Centre Copenhagen, Capital Region of Denmark, Copenhagen University Hospital, Copenhagen, Denmark; 8 Institute of Biological Psychiatry, Mental Health Services, Copenhagen University, Copenhagen, Denmark; 9 Department of Clinical Medicine, University of Copenhagen, Copenhagen, Denmark; 10 Lundbeck Foundation Center for GeoGenetics, GLOBE Institute, University of Copenhagen, Copenhagen, Denmark; 11 Psychosis Research Unit, Aarhus University Hospital Psychiatry, Aarhus, Denmark; 12 NCRR National Centre for Register-Based Research, School of Business and Social Sciences, Aarhus University, Aarhus, Denmark; 13 Centre for Integrated Register-based Research, CIRRAU, Aarhus University, Aarhus, Denmark

**Keywords:** Predictive markers, Psychiatric disorders

## Abstract

Pharmacogenetics aims to improve clinical care by studying the relationship between genetic variation and variable drug response. Large population-based datasets could improve our current understanding of pharmacogenetics from selected study populations. We provide real-world pharmacogenetic frequencies of genotypes and (combined) phenotypes of a large Danish population-based case-cohort sample (iPSYCH2012; data of the Integrative Psychiatric Research consortium). The genotyped sample consists of 77,684 individuals, of which 51,464 individuals had diagnoses of severe mental disorders (SMD case-cohort) and 26,220 were individuals randomly selected from the Danish population (population cohort). Array-based genotype data imputed to 8.4 million genetic variants was searched for a selected pharmacogenetic panel of 42 clinically relevant variants and a *CYP2D6* gene deletion and duplication. We identified 19 of 42 variants. Minor allele frequencies (MAFs) were consistent with previously reported MAFs, and did not differ between SMD cases and population cohorts. Almost all individuals carried at least one genetic variant (> 99.9%) and 87% carried three or more genetic variants. When genotypes were translated into phenotypes, also > 99.9% of individuals had at least one divergent phenotype (i.e. divergent from the common phenotypes considered normal, e.g. extensive metabolizer). The high number of identified individuals with at least one pharmacogenetic variant or divergent phenotype indicates the importance of pharmacogenetic panel-based genotyping. Combined CYP2C19-CYP2D6 phenotypes revealed that 72.7% of individuals had divergent phenotypes for one or both enzymes. As CYP2D6 and CYP2C19 have an important role in the metabolism of psychotropic drugs, this indicates the relevance of pharmacogenetic testing specifically in individuals using psychotropic drugs.

## Introduction

Pharmacogenetics (PGx) studies the relation between genetic variation and variable drug response, with the ultimate aim of improving clinical care^1^. An association between a specific drug and a genetic variant affecting the clinical drug outcomes is referred to as a drug–gene pair or interaction (DGI). Within research, PGx has gained increased attention over the past decades, for example by clinical studies investigating the application of prospective PGx testing to benefit patients’ therapy^2–4^. Now, clinical uptake of PGx testing is improving as well in different clinical areas (e.g. in oncology), but remains modest in the field of psychiatry despite growing evidence that PGx could also benefit the healthcare of psychiatric patients focusing on five PGx genes (cytochrome P450 (CYP) 2C9, 2C19, 2D6, human leukocyte antigen (HLA) -A, -B) linked to DGIs of psychotropics^5,6^.

When PGx tests are used in a clinical care setting mainly single-gene tests are used, either prospectively (i.e. prior to the start of therapy) or retrospectively (i.e. in response to ineffective therapy or adverse events). After performing a PGx test, PGx guidelines direct therapy adjustments in drug users who carry mutant alleles of genetic variants. These mutant alleles are here referred to as ‘actionable PGx variants’. PGx guidelines are provided by international consortia such as the Clinical Pharmacogenetics Implementation Consortium (CPIC) and Dutch Pharmacogenetics Working Group (DPWG) of the Royal Dutch Pharmacist’s Association^7–9^. PGx guidelines are available for a wide range of different drugs from different therapeutic areas, including internal medicine, cardiology, oncology, and psychiatry used in both primary and hospital care. Currently, one-third of the PGx guidelines describe psychotropic drugs, such as the widely used antidepressants citalopram and sertraline^5^.

The interest in panel-based genotyping, instead of single-gene tests, has increased over the past years. Panel-based genotyping include simultaneous testing of multiple genes for which PGx guidelines are available^1^. Panel-based genotyping could increase the clinical usefulness of PGx testing, as it was shown that 91–96% of the individuals of previously studied cohorts carried at least one actionable PGx variant^10–12^. Panel-based genotyping can be executed in response to a first drug prescription, and results can be re-used for all subsequent drug prescriptions in the future, so-called ‘reactive pre-emptive’ PGx testing^13^. With panel-based genotyping, it is possible to use combinatorial PGx, in which multiple variants on different genes are interpreted together to provide the most accurate personal PGx advice, which is especially beneficial if drugs have multiple DGIs or in case of concomitant drug use for which PGx guidelines are available^14,15^.

Several psychotropic drugs, such as amitriptyline or nortriptyline, have PGx guidelines linked to both CYP2C19 and CYP2D6 enzymes^5^, thus increasing the importance of knowing both CYP2C19 and CYP2D6 phenotypes in individuals using these drugs. This also applies to individuals who switch between psychopharmacological therapies which have PGx guidelines for either CYP2C19 or CYP2D6. Investigating frequencies of combinations of CYP2C19-CYP2D6 phenotypes per individual is therefore informative. This also applies to DGIs of non-psychotropics, such as warfarin and VKORC1, CYP2C9, and CYP4F2.

Current associations between PGx geno- or phenotypes and clinical outcomes of drug use are often based on small clinical studies, which limits the generalizability of the results and could slow down the uptake of PGx testing in clinical care^16,17^. Large, unselected, population-based cohorts investigating such associations are currently limited in the literature. Here we make use of a large Danish psychiatry-focused population-based case-cohort sample, which for the first time combines population-based pharmacological treatment data with genotyping data, together with other clinical and socio-demographic characteristics. In a first step, prior to future treatment outcome-focused studies, the aim of the present study is to provide the PGx genotype, phenotype, and combined phenotype frequencies of this large Danish population-based case-cohort sample, based on current clinically relevant PGx variants, aiming to improve our current understanding of PGx of selected (clinical) study populations. We include DGIs of both psychotropics and non-psychotropics in order to investigate complete PGx profiles. In addition, we focus on PGx in psychiatry specifically.

## Materials & methods

### Study design and setting

This study investigates PGx genotype and phenotype profiles of individuals in a large Danish population-based case-cohort sample. This study used data of the Integrative Psychiatric Research (iPSYCH) consortium, which has established a large Danish population-based case-cohort study sample (iPSYCH2012), hereafter referred to as iPSYCH sample^18^. Details on the iPSYCH sample were described previously^18^, but are presented here in brief.

The iPSYCH sample is nested within the entire Danish population born between 1981 and 2005. The iPSYCH sample contains data of two cohorts. The SMD case-cohort (*N* = 57,377) includes all individuals who had one or more diagnoses of five selected severe mental disorders (i.e. affective/mood disorder (depression); attention-deficit/hyperactivity disorder (ADHD); autism spectrum disorder; bipolar affective disorder; and schizophrenia) prior to December 31, 2012. These mental disorders are classified as severe since the individuals had related records of psychiatric hospital contacts and were diagnosed according to the WHO International Classification of Diseases (ICD-10). The population cohort is a random sample of the Danish population of 30,000 individuals. Population individuals could develop one or more SMD (reflecting the normal risk). This sampled cohort is representative of the entire Danish population born in the same period.

### Sample collection and genotyping

The genetic information of the individuals in the iPSYCH sample was collected using dried blood spots retrieved from the Danish Neonatal Screening Biobank within the Danish National Biobank^19^. This facility stored dried blood spot samples taken from practically all neonates born in Denmark between May 1, 1981, and December 31, 2005. Ethnicity was defined based on parental place of birth^18^. The samples were stored for follow-up diagnostics, screening, quality control, and research and kept at −20 °C. At the time of blood sampling (4–7 days after birth), parents were informed in writing about the neonatal screening and that the blood spots are stored in the Danish Neonatal Screening Biobank and can be used for research, pending approval from relevant authorities. The parents were also informed about how to prevent or withdraw the sample from inclusion in research studies.

A total of 80,422 samples were genotyped, as described previously^18^. In brief, whole-genome genotyping was performed with array genotyping (Infinium PsychChip v1.0 array; Illumina, San Diego, CA, USA). After quality control, a total of 77,684 samples remained. Genotyping results have been optimized and imputation of the data has been executed using the 1000 Genomes phase 3 reference panel comprising SNPs (single- nucleotide polymorphisms), INDELs (gene insertions and deletions), and structural variants (unpublished data). This has resulted in a quality-controlled database of about 8.4 million variants over 77,684 individuals, hereafter referred to as iPSYCH genotyped dataset. The genotyping data is linked to Danish medical and sociodemographic registers via a personal identification number, as previously described^18^. Yet, the medical and sociodemographic data will not be presented or analysed in this study.

### PGx genotypes of interest

In order to investigate complete PGx profiles, our analyses are based on a previously defined PGx panel by the ubiquitous-PGx (U-PGx) consortium, including current clinically relevant PGx variants, which include DGIs of both psychotropics and non-psychotropics^20,21^. The original panel was selected to use in a clinical trial implementing pre-emptive genotyping, based on the following criteria: (i) a DPWG guideline with therapeutic recommendations for the specific genotype exists; (ii) the effect of the variant on the gene is established; (iii) the overall minor allele frequency (MAF) is ≥ 1% in the total population or in selected populations; (iv) the variant is already determined in clinical care by one of the implementation sites of the U-PGx study^20^. The authors described the U-PGx panel as a continuously updated panel, which might vary over time, and an updated version of the panel was published in 2019^21^. In this study we use the 2019 U-PGx panel as a reference list of genetic variants which currently have clinical relevance. The panel consists of 42 genetic variants (SNPs), a *CYP2D6* duplication, and *CYP2D6* deletion (Supplementary Table 1). Genetic variants can be described as star allele nomenclatures (*-allele), including one or more specific variants. We checked the Hardy–Weinberg equilibrium (HWE) of the identified variants, a principle stating that genetic variation in a population will remain constant from one generation to the next in the absence of disturbing factors.

### Genotype–phenotype translation

In PGx dosing guidelines, recommendations are often provided based on a specific phenotype rather than genetic variants. Therefore, in this study, the genotypes are translated into phenotypes according to a genotype–phenotype translation table (Supplementary Table 2). Phasing data were not available; thus, we assumed worst-case scenarios when more than one variant was present in the same gene (i.e. variants on different alleles, thus each allele carrying one mutation. An example is described in Supplementary Table 2). For each gene, two haplotypes represent a diplotype, which is translated into a phenotype. Phenotypes are generally listed as poor metabolizers (PM), intermediate metabolizers (IM), normal or extensive metabolizers (EM), and (ultra)rapid metabolizers (UM). Some exceptions have been described differently, such as CYP3A5 phenotypes described as non-expressers, heterozygous expressers and homozygous expressers, dihydropyrimidine dehydrogenase (*DPYD*) phenotypes as a gene activity score (GAS) ranging from 0 to 2, factor V Leiden (FVL) phenotypes as wild-type (WT), heterozygous carrier or homozygous carrier, vitamin K epoxide reductase complex 1 (VKORC1) phenotypes as high or normal sensitivity. CYP3A5 homozygous expressers, DPYD GAS 2, FVL wild-type, and VKORC1 normal sensitivity refer to extensive metabolic capacity and normal protein function. An example of a genotype–phenotype translation is described. A person is the carrier of a single variant of rs1799853 in *CYP2C9*. This person is then listed as *2 haplotype, *1/*2 diplotype, and IM phenotype for *CYP2C9*. The translation table is created according to current CPIC and DPWG guidelines. DPWG guidelines were prioritised in case of disagreement between guidelines from CPIC and DPWG, with the exception of *CYP2C19* *1/*17 classified as rapid metabolizer (RM) according to CPIC guidelines. A consensus between CPIC and DPWG was recently accomplished for the *CYP2D6* genotype-phenotype translation^22^.

### Data accessibility

The iPSYCH study was approved by the Danish Scientific Ethics Committee (EC: 1-10-72-287-12), the Danish National Board of Health (Sundhedsdatastyrelsen, SDS, FSEID 1999), the Danish Data Protection Agency (Journal number 2015-57-0002, 62908, umbrella permission Aarhus University), and the Danish Neonatal Screening Biobank Steering Committee. All data are stored at Statistics Denmark and were available in an anonymous form, by remote online access, with special permission in compliance with the Danish Data Privacy Act.

### Statistical analyses

The allele frequency was calculated for the total sample and the SMD case and population cohorts. We report genotype and phenotype frequencies, and combined phenotype frequencies of CYP2C19-CYP2D6 (PGx guidelines for psychotropic drugs) and VKORC1-CYP2C9 (PGx guidelines for warfarin). In this study we refer to ‘normal’ phenotypes as the phenotypes with an extensive metabolic capacity and normal protein function, which are often found in the majority of the population and on which drug prescription guidelines are based (here EM, homozygous expressers, WT or *DPYD* GAS 2). Thus, in this study, the divergent phenotypes are the phenotypes that are not EM, homozygous expressers, WT, or *DPYD* GAS 2. We did not distinguish between specific drug users in our calculations (e.g. FVL variant is relevant for users of oral contraceptives, which are females).

Due to a restriction from Statistics Denmark and the General Data Protection Regulations, data can only be reported if the number of individuals contributing to aggregated measures exceeds four, which can result in the grouping of data. All data processing and analyses were conducted in R version 3.4.2 (R Core Team, 2017)^23^.

## Results

### Characteristics of the iPSYCH genotyped dataset

Of the total iPSYCH sample of 87,377 individuals, including 57,377 individuals with SMD and 30,000 population individuals without SMD, we identified 77,684 individuals with valid genotype data in total, split into the SMD case-cohort (51,464 individuals with SMD) and the population cohort (26,220 population individuals, Table 1). The SMD case-cohort had more males (54.7%) compared to the population cohort (51%), linked to a higher number of individuals with autism and ADHD in the SMD case-cohort. The vast majority of the cohort had European ethnic backgrounds (including Denmark, other Scandinavian countries, and other European countries). The SMD case-cohort consisted mostly of individuals with depression, ADHD, and autism and less of individuals with schizophrenia and bipolar affective disorder, following the iPSYCH sample^18^.

**Table 1:** Characteristics of the sample.

	Total sample		SMD case-cohort		Population cohort	
	(*N* = 77,684)		(*N* = 51,464)		(*N* = 26,220)	
	*N*	(%)	*N*	(%)	*N*	(%)
*Sex*						
Female	36,155	(46.5)	23,309	(45.3)	12,846	(49.0)
Male	41,529	(53.5)	28,155	(54.7)	13,374	(51.0)
*Ethnicity*^*a*^						
Africa	470	(0.6)	234	(0.5)	236	(0.9)
Asia	588	(0.8)	263	(0.5)	325	(1.2)
Australia/Greenland/N. & S. America/Unknown	49	(0.06)	26	(0.05)	23	(0.09)
Denmark	66,810	(86.0)	44,696	(86.8)	22,114	(84.3)
Europe	1163	(1.5)	599	(1.2)	564	(2.2)
Middle East	773	(1.0)	376	(0.7)	397	(1.5)
Mixed	7722	(9.9)	5217	(10.1)	2505	(9.6)
Scandinavia	109	(0.1)	53	(0.1)	56	(0.2)
*Diagnosis with SMD*^*b*^						
ADHD	17,004	(21.9)	16,646	(32.3)	358	(1.4)
Bipolar affective disorder	1419	(1.8)	1388	(2.7)	31	(0.1)
Depression	22,374	(28.8)	21,914	(42.6)	460	(1.8)
Autism spectrum disorder	14,602	(18.8)	14,293	(27.8)	309	(1.2)
Schizophrenia	2685	(3.5)	2620	(5.1)	65	(0.2)

*SMD* severe mental disorder, *ADHD* attention deficit hyperactivity disorder.

^a^*Ethnicity* was defined based on parental place of birth as described by Pedersen et al^18^. Europe means other countries than Denmark/Scandinavia and Scandinavia means only Norway, Sweden, Finland, and Iceland. If one parent was born outside of Denmark, that region was used. If both parents were born in different regions outside of Denmark, mixed was used.

^b^Summing the percentages of individual disorders might add up to more than 100%, as persons can carry multiple diagnoses.

Presented are characteristics of individuals from the iPSYCH2012 sample as a total sample, and a separate SMD case-cohort and population cohort. This updated table includes only genotyped individuals and differs slightly from the table with characteristics published by Pedersen et al.^18^.

### PGx genotypes

The reference PGx panel consisted of 42 clinically relevant variants, a *CYP2D6* duplication, and deletion (Supplementary Table 1)^20,21^. In the imputed iPSYCH genotyped dataset 19 out of 42 variants were identified with fully available data. Supplementary Table 3 shows the identified variants, the MAFs from literature for these variants, and MAFs identified in this sample^24^. Similar MAFs were found between the SMD case-cohort and the population cohort. MAFs were consistent with previously reported MAFs in European-based populations. The sample consisted of multiple ethnicities (thus including population migrations) and HWE was not reached for each variant in each cohort (using chi-squared test, Supplementary Table 3). Yet, HWE tests are expected to perform poorly in mixed ethnicity samples. The variants with a deviating HWE were checked in individuals with a Danish ethnic background only, resulting in HWE for each variant except rs67376798.

**Fig. 1:**
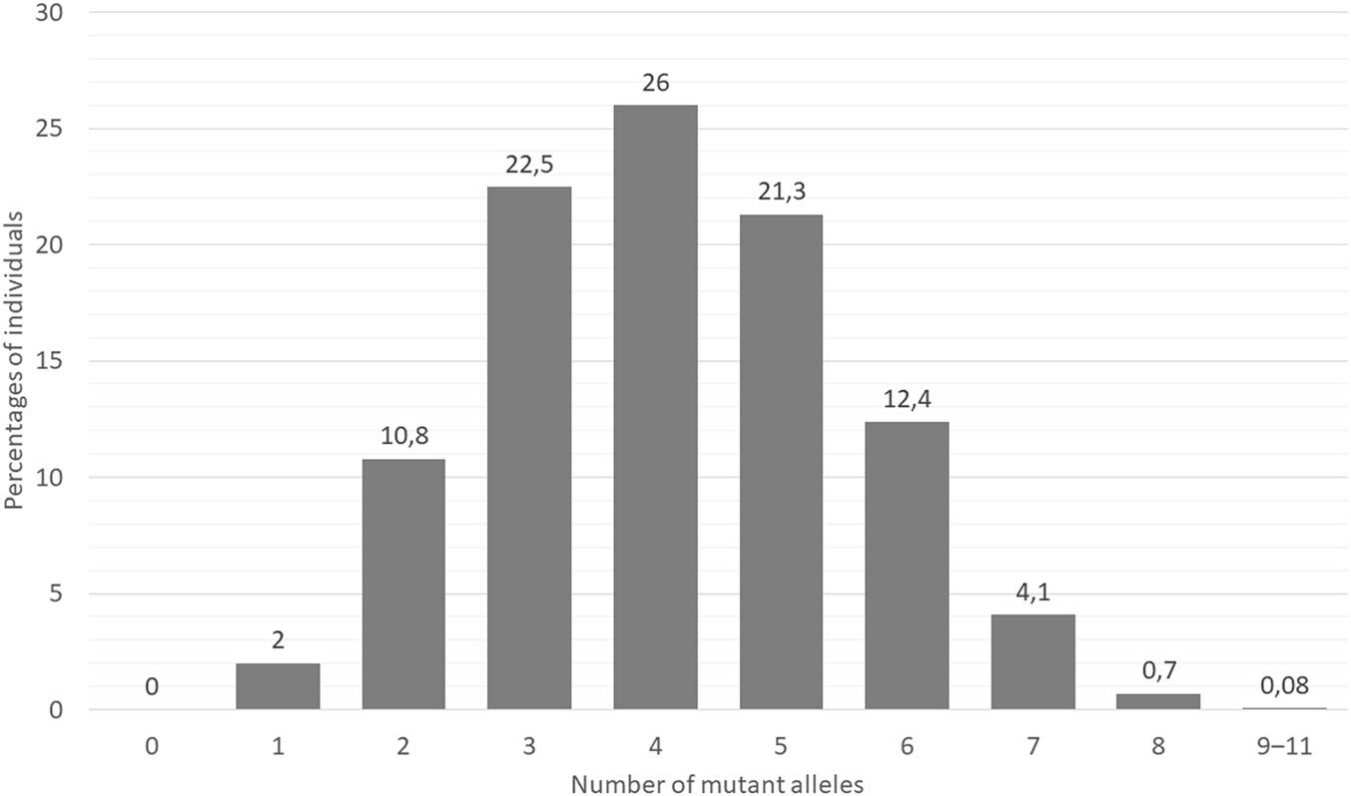
Individuals with different numbers of actionable PGx variants in the sample. Shown are the percentages of individuals from the total sample who carry different numbers of actionable PGx variants. PGx pharmacogenetics.

The number of actionable PGx variants per individual showed that almost every individual (> 99.9%) carried at least one actionable PGx variant of the 19 clinically relevant PGx variants (Fig. 1 and Supplementary Table 4). Up to 98% of the individuals carried two or more clinically relevant PGx variants, and 87% of the individuals carried three or more. Roughly two-thirds of the individuals carried three to five PGx variants out of 19 clinically relevant PGx variants. The numbers of actionable PGx variants per individual were similar in the total sample, SMD case-cohort, and population cohort.

The PGx variants not identified in our data included mostly rare variants. For example, in *CYP2C9* we do not have data of three variants with summed European MAFs of 0.0028 and in *CYP2C19* European MAFs of seven missing variants add up to only 0.016. However, two missing variants in *CYP2B6* had MAFs in European populations of 0.22 and 0.11. In *CYP2D6*, missing variants have summed MAFs of 0.04, yet missing gene deletions and duplications occur more frequently up to 12% in Caucasians^25^. Other incomplete genes with missing summed MAFs were *CYP3A5* (0.0004), *DPYD* (0.019), *HLA-B* (0.036), *SLCO1B1* (0.157), *TPMT* (0.037), and *UGT1A1* (0.30).

### PGx phenotypes

All available genotypes were translated into phenotypes according to the genotype–phenotype translation table (Supplementary Table 2). The frequencies of the identified phenotypes are shown in Table 2. Again, the observed frequencies were similar between the total sample, SMD case-cohort, and the population cohort. The phenotypes EM, WT, and *DPYD* GAS 2 were the most frequent phenotypes within an enzyme. In general, CYP enzymes showed lower frequencies of the phenotype EM, compared to the non-CYP enzymes. In CYP3A5 the vast majority (86%) of the individuals had the non-expresser (no metabolic capacity) phenotype.

**Table 2:** Phenotype frequencies.

Phenotypes	Total sample (*N* = 77,684)		SMD case-cohort (*N* = 51,464)		Population cohort (*N* = 26,220)	
	*N*	(%)	*N*	(%)	*N*	(%)
*CYP2B6*						
EM	45,177	(58.2)	30,048	(58.4)	15,129	(57.7)
IM	27,949	(36.0)	18,439	(35.8)	9510	(36.3)
PM	4558	(5.9)	2977	(5.8)	1581	(6.0)
*CYP2C9*						
EM	51,612	(66.4)	34,094	(66.3)	17,518	(66.8)
IM	23,347	(30.1)	15,542	(30.2)	7805	(29.8)
PM	2725	(3.5)	1828	(3.6)	897	(3.4)
*CYP2C19*						
UM	2943	(3.8)	1914	(3.7)	1029	(3.9)
RM	20,048	(25.8)	13,279	(25.8)	6769	(25.8)
EM	33,856	(43.6)	22,386	(43.5)	11,470	(43.8)
IM	19,118	(24.6)	12,761	(24.8)	6357	(24.2)
PM	1719	(2.2)	1124	(2.18)	595	(2.3)
*CYP2D6*						
EM	48,455	(62.4)	32,097	(62.4)	16,358	(62.4)
IM	25,980	(33.4)	17,232	(33.5)	8748	(33.4)
PM	3249	(4.2)	2135	(4.2)	1114	(4.3)
*CYP3A5*						
Homozygote expresser	490	(0.6)	310	(0.6)	180	(0.7)
Heterozygote expresser	10,161	(13.1)	6587	(12.8)	3574	(13.6)
Non-expresser	67,033	(86.3)	44,567	(86.6)	22,466	(85.7)
*DPYD*						
GAS 2	76,144	(98.0)	50,425	(98.0)	25,719	(98.1)
GAS 1.5	535	(0.7)	354	(0.7)	181	(0.7)
GAS 1	<1000	(<1.3)	<690	(<1.4)	<320	(<1.3)
GAS 0.5	<5	(<0.01)	<5	(<0.02)	<5	(<0.03)
GAS 0	<5	(<0.01)	<5	(<0.02)	<5	(<0.03)
*FVL*						
WT	72,453	(93.3)	47,989	(93.3)	24,464	(93.3)
Heterozygous carrier	5141	(6.6)	3410	(6.6)	1731	(6.6)
Homozygous carrier	90	(0.1)	65	(0.1)	25	(0.1)
*TPMT*						
EM	70,034	(90.2)	46,401	(90.2)	23,633	(90.1)
IM	7429	(9.6)	4918	(9.6)	2511	(9.6)
PM	221	(0.3)	145	(0.3)	76	(0.3)
*UGT1A1*						
EM	77,229	(99.4)	51,177	(99.4)	26,052	(99.4)
IM	447	(0.6)	<290	(<0.6)	<170	(<0.7)
PM	8	(0.01)	<5	(<0.01)	<5	(<0.02)
*VKORC1*						
WT (NS)	36,831	(47.4)	24,412	(47.4)	12,419	(47.4)
Heterozygous carrier (NS)	28,861	(37.2)	19,166	(37.2)	9695	(37.0)
Homozygous carrier (HS)	11,992	(15.4)	7886	(15.3)	4106	(15.7)

*SMD* severe mental disorders, *EM* extensive metabolizer, *IM* intermediate metabolizer, *PM* poor metabolizer, *UM* ultrarapid metabolizer, *RM* rapid metabolizer, *DPYD* dihydropyrimidine dehydrogenase, *GAS* gene activity score, *FVL* factor V Leiden, *WT* wild-type carrier, *TPMT* thiopurine methyltransferase, *UGT1A1* UDP-glucuronosyltransferase 1A1, *VKORC1* vitamin K epoxide reductase complex 1, *NS* normal sensitivity, *HS* high sensitivity.

Numbers with < sign were slightly adjusted to prevent the possibility of calculating the number of individuals < 5.

Phenotype distributions of the total sample and for the SMD case-cohort and population cohort based on the available genotypes.

As we investigated the number of actionable PGx variants per individual, we also investigated the number of divergent phenotypes per individual (i.e. not EM, homozygous expressers, WT or *DPYD* GAS 2, Table 3). Similar results to the actionable PGx variant distributions were identified, with > 99.9% of the individuals having one or more divergent phenotypes. Up to 97% of the individuals have two or more divergent phenotypes, and 82% of the individuals have three or more divergent phenotypes. Roughly two-thirds of the individuals carried three or four divergent phenotypes. The numbers of divergent phenotypes per individual were similar in the total sample, SMD case-cohort, and population cohort (Table 3).

**Table 3:** Number of divergent phenotypes per individual in the sample.

Number of divergent phenotypes	Total sample (*N* = 77,684)		SMD case-cohort (*N* = 51,464)		Population cohort (*N* = 26,220)	
	*N*	(%)	*N*	(%)	*N*	(%)
0	21	(0.03)	12	(0.02)	9	(0.03)
1	2014	(2.6)	1 348	(2.6)	666	(2.5)
2	11,764	(15.1)	7804	(15.2)	3960	(15.1)
3	25,623	(33.0)	16,931	(32.9)	8692	(33.2)
4	24,226	(31.2)	16 046	(31.2)	8180	(31.2)
5	11,294	(14.5)	7513	(14.6)	3781	(14.4)
6	2461	(3.2)	1628	(3.2)	833	(3.2)
7–9^a^	281	(0.4)	182	(0.4)	99	(0.4)

*SMD* severe mental disorders, *DPYD* dihydropyrimidine dehydrogenase, *GAS* gene activity score, *FVL* factor V Leiden, *TPMT* thiopurine methyltransferase, *UGT1A1* UDP-glucuronosyltransferase 1A1, *VKORC1* vitamin K epoxide reductase complex 1.

^a^Numbers in this row were grouped to prevent showing data <5.

Ten phenotypes (CYP2B6, CYP2C9, CYP2C19, CYP2D6, CYP3A5, DPYD, FVL, TPMT, UGT1A1, VKORC1) are included. Divergent phenotypes are considered phenotypes that are not extensive metabolizers, homozygote expressers, DPYD GAS 2, or wild-type carriers.

### Combined phenotypes

**Fig. 2:**
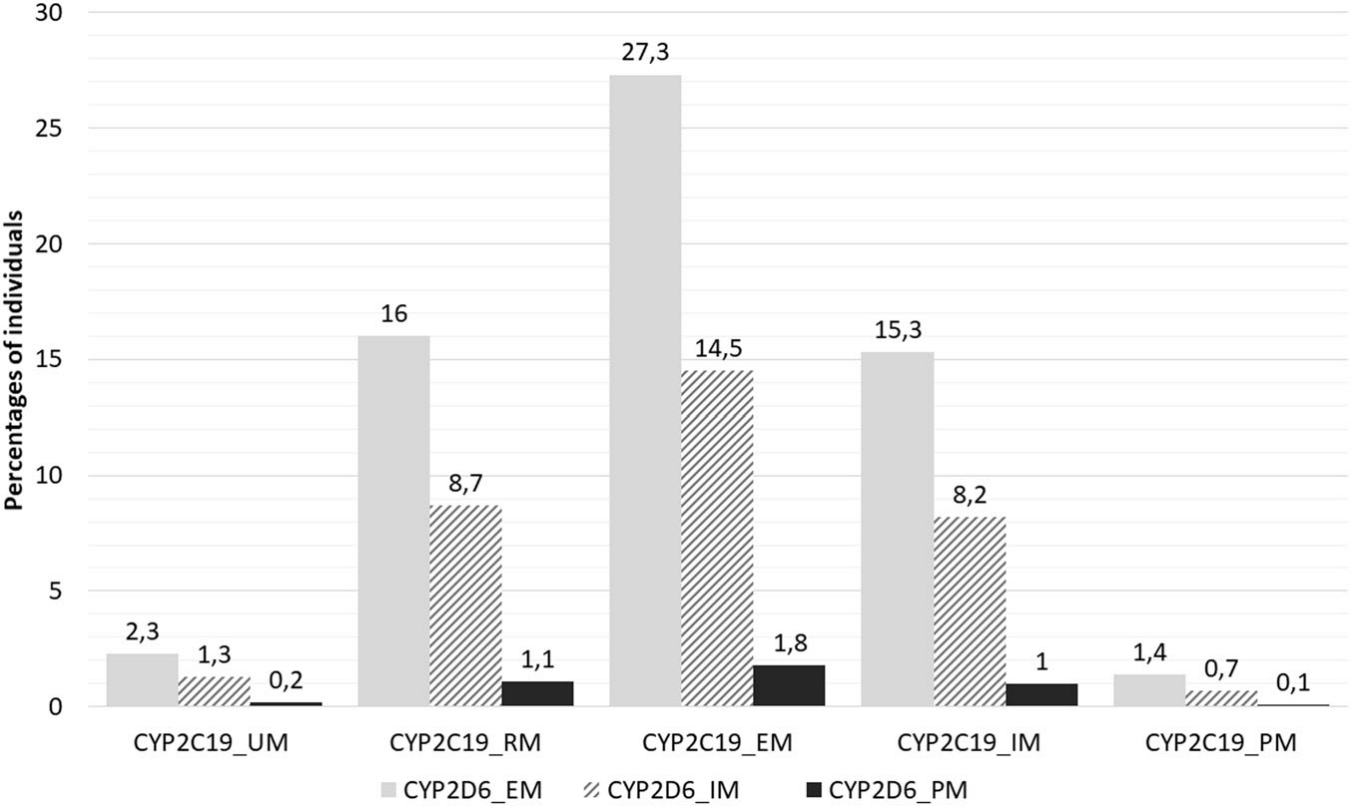
Individuals with CYP2C19-CYP2D6 combined phenotypes. Shown are the percentages of individuals from the total sample having CYP2C19-CYP2D6 combined phenotypes (Supplementary Table 5). For example, 27.3% of the individuals have the combination of CYP2C19 EM and CYP2D6 EM phenotypes, and 8.2% of the individuals have CYP2C19 IM combined with CYP2D6 IM phenotype status. EM extensive metabolizer; IM intermediate metabolizer; UM ultrarapid metabolizer; RM rapid metabolizer; PM poor metabolizer.

The largest group of CYP2C19-CYP2D6 combined phenotypes are individuals with CYP2C19 EM-CYP2D6 EM (27%, Fig. 2), indicating that a majority (73%) of the individuals has a divergent phenotype for either CYP2C19 or CYP2D6 or both, and thus having a likely chance of requiring a therapy adjustment when treated with one or more psychotropic drugs for which PGx guidelines are available. A small subgroup of individuals (10%) has phenotypes CYP2C19 IM/PM-CYP2D6 IM/PM. For the phenotype combination VKORC1-CYP2C9, users of the drug warfarin might need therapy adjustments from current PGx guidelines. This includes 5% of the individuals who have divergent phenotypes for both VKORC1 and CYP2C9 and 29% of individuals with only divergent phenotypes for CYP2C9. The observed frequencies of the total sample, SMD case-cohort, and the population cohort were similar (Supplementary Table 5).

## Discussion

In this study, we investigated the PGx genotype and phenotype profiles of 77,684 individuals in a large Danish population-based case-cohort sample. We report genotype and translated phenotype frequencies and combined phenotype frequencies of CYP2C19-CYP2D6 and CYP2C9-VKORC1.

We identified 19 clinically relevant PGx variants and found that > 99.9% of individuals from this population-based sample carried at least one actionable PGx variant. This corresponds to previously reported results where 91–96% of individuals in selected study populations carried at least one actionable PGx variant^10–12^. The minor differences between our results and previously reported results might be due to ethnic variability or differences in the investigated (number of) variants in those studies. More importantly, still 87% of the population individuals in our study were carriers of three or more clinically relevant actionable PGx variants. Important combinations of phenotypes are CYP2C19-CYP2D6 and VKORC1-CYP2C9, as there are PGx drugs having guidelines for both enzymes. 10% of the individuals had an IM or PM phenotype for both CYP2C19 and CYP2D6, and therefore have a higher risk of developing adverse events from psychotropic drug treatment for which PGx guidelines are available if therapy adjustments are not applied. These numbers show the relevance of panel-based testing compared to single-gene testing, as it is likely multiple actionable PGx variants can be identified within one individual. The genetic data in this study is linked to pharmacological treatment data, in contrast to the previously genotyped study populations^10–12^, and this sample will be used for future research investigating PGx. A study investigating drug utilization patterns of psychotropic PGx drugs in SMD cohorts and the population cohort is currently submitted (Lunenburg et al.). The study includes the proportion of individuals who have drug choice or drug dosing recommendations based on CPIC and DPWG guidelines classified as actionable according to their CYP2D6 and CYP2C19 divergent genotype–phenotypes.

To the best of our knowledge, we are not aware of any association between the investigated variants and an increased risk for onset of SMD. Our results confirm this, as MAFs did not differ between the cohorts. Even though the results were very similar, we have chosen to display the cohorts separately in the tables to support referencing in future studies specifically for individuals with SMD or population-based individuals. The previously reported characteristics of the iPSYCH2012 sample^18^ and the characteristics in this study differ slightly, possibly due to including only individuals with fully available genotypes, slight differences in a grouping of individuals, or updated data. The fact that relatively more males are present in the SMD case-cohort is because more individuals with autism and ADHD are present in this cohort, which are psychiatric disorders especially prevalent in young males^26,27^.

The vast majority of individuals had one or more divergent phenotypes and might require therapy adjustments based on PGx guidelines^7–9^. Of course, PGx guidelines can only be applied to individuals with a specific geno- or phenotype who start using the related drug for which the PGx guidelines are provided. Yet, when individuals have multiple actionable PGx variants or divergent phenotypes, the chance of them starting the related drug for which PGx guidelines are available increases. The similarity in the genotype and phenotype results might indicate that individuals who carry multiple variants, carry these variants in different genes. In our calculations, we included male carriers of the PGx variant in FVL, even though they will not use oral contraceptives. We do not expect this to result in a significant bias of our results as the FVL variant has a low frequency.

The presentation of genotype and (combined) phenotype frequencies of a large population-based sample of over 77,000 individuals is a strength of this study. The study includes individuals with SMD and a randomly selected population cohort, which is representative of the entire Danish population of young age. The sample has no bias in the selection of individuals, little loss to follow-up, and consistent data collection through several Danish registries. Yet, there are also some limitations in this study. Ideally, we would have translated the predicted phenotypes using all clinically relevant variants, but we only identified 19 out of 42 PGx variants. We had not expected to identify all 42 variants as we used a preselected psychiatry-focused genotyping array. There are multiple explanations possible for the limited number of identified variants. It could be linked to the quality of the genotyping, the MAF of the missing variants (i.e. rare variants), patterns in linkage disequilibrium of certain variants, or that the assay used to genotype the sample was a whole-genome genotyping array suitable for large-scale genetic studies focused on psychiatric predisposition and risk, not on PGx related variants. The 42 variants are located in different genes and are mostly linked to drug metabolism, not psychiatric predisposition. All variants with a MAF below 1% were excluded in the executed imputation of the data, improving the trustworthiness of the imputed variants, but this also contributes to the limited identification of variants with a low MAF. In addition, we did not have phasing data available in this study, and we assumed worst-case scenarios in the genotype-phenotype translations. Lastly, we used imputed data based on a selected array for research purposes, which cannot be used to identify these PGx variants in daily clinical care.

The unavailability of data on rare variants in this study results in a limited bias towards the phenotype frequency predictions. In contrast, some of the missing variants were not rare variants. If data on all 42 variants would have been available, we would expect to find higher numbers of different PGx variants per individual, or possibly more deviant phenotypes in individuals when the identified variants per individual were located in the same genes. However, it is tricky to calculate this bias in the phenotype predictions from the missing variants in this study. The individual variant MAFs are known, but how frequently they occur together in different combinations varies between studies depending on the included variants. Average reported phenotype frequencies may therefore differ from ours. In addition, the allocation of specific alleles to a phenotype status may differ between the consortia providing PGx guidelines, adding to different reported phenotype frequencies in previous studies. An example is CYP2C19 *1/*17, which is considered EM by DPWG, but rapid metabolizer (RM) by CPIC^28,29^. Yet, the RM phenotype follows the same recommendations as UM in CPIC guidelines, e.g. for tricyclic antidepressants^30^. Another example is CYP2C9 *2/*2, which is classified as PM by DPWG and IM by CPIC.

In conclusion, we have provided real-world population-based knowledge on frequencies of PGx genotypes and phenotypes. Assuming PGx testing benefits patients’ healthcare, the high number of identified individuals with at least one PGx variant or divergent phenotype indicates the importance of PGx panel-based genotyping. We have seen a greater variability in phenotypes concerning CYP enzymes compared to non-CYP enzymes, yet we consider all to be important as part of the clinically relevant genotyping panel with PGx guidelines available. The high frequency of combined divergent phenotypes of CYP2C19 and CYP2D6 indicates the relevance of PGx testing specifically in individuals using psychotropic drugs.

## Supplementary information

Supplement material
