# Supplementary material for: Pharmacogenetic genotype and phenotype frequencies in a large Danish population-based case-cohort sample
Source: Transl Psychiatry. 2021 May 18;11:294. doi: 10.1038/s41398-021-01417-4 (PMC8131614; doi:10.1038/s41398-021-01417-4)
Supplement: Supplementary file 1 — Supplement material [file 41398_2021_1417_MOESM1_ESM.docx]

**Supplement to:**

Pharmacogenetic genotype and phenotype frequencies in a large Danish population-based case-cohort sample.

Lunenburg *et al.*

Supplement Table 1: List of clinically relevant PGx variants

The table shows 42 clinically relevant PGx variants, a CYP2D6 gene duplication and a CYP2D6 gene deletion. We used this table as a reference table and searched for these variants. The table lists the gene name, related RS-number of the variant, the linked *-allele nomenclature and the genomic position (chromosome, base pair position and nucleotide change) according to Genome Reference Consortium Human Build 37 (GRCh37). The genetic variants of the PGx panel are so-called 'key variants', meaning they are the key defining variant for a *-allele.(1,2) In some cases the same *-allele is listed at several variants, e.g. *CYP2D6* rs3892097 and rs1065852. Both variants together label the haplotype as *CYP2D6*4*.

| **Gene/protein** | **RS-number** | **Allele nomenclature** | **Genomic position (Chr, bp, nucleotide change)** | | |
| --- | --- | --- | --- | --- | --- |
| CYP2B6 | rs2279343 | *4/*6/*16 | 19 | 41515263 | A>G |
| CYP2B6 | rs3211371 | *5 | 19 | 41522715 | C>T |
| CYP2B6 | rs3745274 | *6/*9 | 19 | 41512841 | G>T |
| CYP2B6 | [rs28399499](https://www.ncbi.nlm.nih.gov/snp/rs28399499) | *16/*18 | 19 | 41518221 | T>C |
| CYP2C19 | rs4244285 | *2 | 10 | 96541616 | G>A |
| CYP2C19 | rs4986893 | *3 | 10 | 96540410 | G>A |
| CYP2C19 | rs28399504 | *4A/B | 10 | 96522463 | A>G |
| CYP2C19 | rs56337013 | *5 | 10 | 96612495 | C>T |
| CYP2C19 | rs72552267 | *6 | 10 | 96535210 | G>A |
| CYP2C19 | rs41291556 | *8 | 10 | 96535173 | T>C |
| CYP2C19 | rs17884712 | *9 | 10 | 96535246 | G>A |
| CYP2C19 | rs6413438 | *10 | 10 | 96541615 | C>T |
| CYP2C19 | rs12248560 | *17 | 10 | 96521657 | C>T |
| CYP2C9 | rs1799853 | *2 | 10 | 96702047 | C>T |
| CYP2C9 | rs1057910 | *3 | 10 | 96741053 | A>C |
| CYP2C9 | rs28371686 | *5 | 10 | 96741058 | C>G |
| CYP2C9 | rs28371685 | *11 | 10 | 96740981 | C>T |
| CYP2D6 | X | xN duplication | 22 | X | X |
| CYP2D6 | X | *5 / deletion | 22 | X | X |
| CYP2D6 | rs35742686 | *3 | 22 | 42524244 | delT |
| CYP2D6 | rs3892097 | *4 | 22 | 42524947 | C>T |
| CYP2D6 | rs1065852 | *4/*10/*14A | 22 | 42526694 | G>A |
| CYP2D6 | rs5030655 | *6 | 22 | 42525086 | delA |
| CYP2D6 | rs5030865 | *8/*14A/*14B | 22 | 42525035 | C>A/T |
| CYP2D6 | rs5030656 | *9 | 22 | 42524176 | delCTT |
| CYP2D6 | rs28371706 | *17 | 22 | 42525772 | G>A |
| CYP2D6 | rs28371725 | *41 | 22 | 42523805 | C>T |
| CYP3A5 | rs776746 | *3 | 7 | 99270539 | C>T |
| CYP3A5 | rs10264272 | *6 | 7 | 99262835 | C>T |
| CYP3A5 | rs41303343 | *7 | 7 | 99250393_99250394 | insA |
| DPYD | rs3918290 | *2A | 1 | 97915614 | C>T |
| DPYD | rs55886062 | *13 | 1 | 97981343 | A>C |
| DPYD | rs56038477 | (c.1236G>A) | 1 | 98039419 | C>T |
| DPYD | rs67376798 | (c.2846A>T) | 1 | 97547947 | T>A |
| FVL | rs6025 | (c.1691G>A) | 1 | 169519049 | T>C |
| HLA-B | rs2395029 | *5701 | 6 | 31431780 | T>G |
| SLCO1B1 | rs4149056 | *5/*15/*17 | 12 | 21331549 | T>C |
| TPMT | rs1800462 | *2 | 6 | 18143955 | C>G |
| TPMT | rs1800460 | *3A/*3B | 6 | 18139228 | C>T |
| TPMT | rs1142345 | *3A/*3C | 6 | 18130918 | T>C |
| UGT1A1 | rs4148323 | *6 | 2 | 234669144 | G>A |
| UGT1A1 | rs35350960 | *27 | 2 | 234669619 | C>A |
| UGT1A1 | rs8175347 | *28[7]/*37[8] | 2 | 234668881_234668882 | TA[7/8] |
| VKORC1 | rs9934438^1^ | (c.1173C>T) | 16 | 31104878 | G>A |

*^1^ In linkage disequilibrium (LD) with rs9923231 (-1639G>A)*

*Abbreviations: PGx: pharmacogenetics; Chr: chromosome; bp: base pair; del: deletion; ins: insertion; DPYD: dihydropyrimidine dehydrogenase; FVL: factor V Leiden; WT: wild-type carrier; TPMT: thiopurine methyltransferase; UGT1A1: UDP-glucuronosyltransferase 1A1; VKORC1: vitamin K epoxide reductase complex 1.*

Supplement Table 2: Genotype-phenotype translation of available variants

The 19 available PGx variants in this study are presented by predicted diplotypes and phenotypes. As there is no phasing data available, the worst-case-scenario was assumed when an individual was heterozygous for two mutations in one gene, thus each allele carrying one mutation. For example, when a patient is carrier of both one CYP2C19*2 and one CYP2C19*8 variant, we assumed these variants to be on two different alleles (resulting in a CYP2C19*2/CYP2C19*8 genotype (PM), also known as compound heterozygous *in trans)*, rather than these variants to be on the same allele (resulting in a CYP2C19*1/CYP2C19*2+*8 genotype (IM), also known as compound heterozygous *in cis*).

All translations are based on both DPWG and CPIC. DPWG guidelines were prioritised in case of disagreement between guidelines from CPIC and DPWG. Yet, *CYP2C19* *1/*17 (RM) was classified according to CPIC. *CYP2B6* *1/*9 (IM) and *CYP2B6* *9/*9 (PM) were classified based on CPIC, however, DPWG recommends *CYP2B6* *1/*6 (IM) and *CYP2B6* *6/*6 (PM) and these *-alleles share the same genetic variant and another variant in linkage disequilibrium.

| ***Gene*** | ***Predicted diplotype*** | ***Phenotype*** |
| --- | --- | --- |
| CYP2B6 | *1/*1 | EM |
| CYP2B6 | *1/*9 | IM |
| CYP2B6 | *9/*9 | PM |
| CYP2C9 | *1/*1 | EM |
| CYP2C9 | *1/*2, *1/*3 | IM |
| CYP2C9 | *2/*2, *2/*3, *3/*3 | PM |
| CYP2C19 | *17/*17 | UM |
| CYP2C19 | *1/*17 | RM |
| CYP2C19 | *1/*1 | EM |
| CYP2C19 | *1/*2, *1/*8, *2/*17 | IM |
| CYP2C19 | *2/*2,*2/*8, *8/*8 | PM |
| CYP2D6 | *1/*1 | GAS 2 (extensive metabolic capacity) |
| CYP2D6 | *1/*17, *1/*41 | GAS 1.5 |
| CYP2D6 | *1/*10 | GAS 1.25 |
| CYP2D6 | *1/*4, *17/*17, *17/*41, *41/*41 | GAS 1 |
| CYP2D6 | *10/*17, *10/*41 | GAS 0.75 |
| CYP2D6 | *4/*17, *4/*41, *10/*10 | GAS 0.5 |
| CYP2D6 | *4/*10 | GAS 0.25 |
| CYP2D6 | *4/*4 | GAS 0 |
| CYP3A5 | *1/*1 | Homozygote expresser (extensive metabolic capacity) |
| CYP3A5 | *1/*3, *1/*6 | Heterozygote expresser (reduced metabolic capacity) |
| CYP3A5 | *3/*3, *3/*6, *6/*6 | Non-expresser (no metabolic capacity) |
| FVL | *1/*1 | WT |
| FVL | *1/rs6025 | Heterozygous carrier |
| FVL | rs6025/rs6025 | Homozygous carrier |
| TPMT | *1/*1 | EM |
| TPMT | *1/*2, *1/*3C | IM |
| TPMT | *2/*2, *2/*3C, *3C/*3C | PM |
| UGT1A1 | *1/*1 | EM |
| UGT1A1 | *1/*6 | IM |
| UGT1A1 | *6/*6 | PM |
| VKORC1 | *1/*1 | WT (normal sensitivity) |
| VKORC1 | *1/c.1173 | Heterozygous carrier (normal sensitivity) |
| VKORC1 | c.1173/c.1173 | Homozygous carrier (high sensitivity) |
| DPYD | *1/*1 | GAS 2 (extensive metabolic capacity) |
| DPYD | *1/c.2846 | GAS 1,5 |
| DPYD | *1/*2A, c.2846/c.2846 | GAS 1 |
| DPYD | *2A/c.2846 | GAS 0,5 |
| DPYD | *2A/*2A | GAS 0 |

*Abbreviations: PGx: pharmacogenetics; DPWG: Dutch Pharmacogenetics Working Group; CPIC: Clinical Pharmacogenetics Implementation Consortium; RM: rapid metabolizer; IM: intermediate metabolizer; PM: poor metabolizer; EM: extensive metabolizer; GAS: gene activity score; WT: wild-type.*

Supplement Table 3: Minor allele frequencies of identified variants in the sample

Shown are the genetic variants identified in the sample, including the MAFs reported at the NCBI of global and European populations and MAFs identified in the total sample and for the population cohort and SMD case cohort separate. Genomic position includes the chromosome number, the base pair position and nucleotide change of the variant.

| **Gene** | **RS-number** | **Allele nomenclature** | **Genomic position** | **Global MAF** | **European MAF** | **Total sample MAF**  **(N=77,684)** | **SMD casecohort MAF**  **(N=51,464)** | **Population cohort MAF**  **(N=26,220)** |
| --- | --- | --- | --- | --- | --- | --- | --- | --- |
| CYP2B6 | rs3745274 | *6, *9 | 19.9:g.41512841G>T | 0.2595 | 0.2417 | 0.2386* | 0.2370* | 0.2416 |
| CYP2C9 | rs1799853 | *2 | 10.10:g.96702047C>T | 0.1128 | 0.1192 | 0.1217 | 0.1229 | 0.1194 |
| CYP2C9 | rs1057910 | *3 | 10.10:g.96741053A>C | 0.0681 | 0.0691 | 0.0637* | 0.0636* | 0.0637 |
| CYP2C19 | rs4244285 | *2 | 10.10:g.96541616G>A | 0.1484 | 0.1489 | 0.1432* | 0.1437 | 0.1423* |
| CYP2C19 | rs41291556 | *8 | 10.10:g.96535173T>C | 0.0028 | 0.0029 | 0.0019 | 0.0021 | 0.0017 |
| CYP2C19 | rs12248560 | *17 | 10.10:g.96521657C>T | 0.2198 | 0.2225 | 0.1951 | 0.1944 | 0.1966 |
| CYP2D6 | rs3892097 | *4 | 22.10:g.42524947C>T | 0.1839 | 0.1928 | 0.2041 | 0.2045 | 0.2034 |
| CYP2D6 | rs1065852 | *4, *10, *14A | 22.10:g.42526694G>A | 0.2123 | 0.2153 | 0.2200 | 0.2194 | 0.2212 |
| CYP2D6 | rs28371706 | *17 | 22.10:g.42525772G>A | 0.0288 | 0.0037 | 0.0023* | 0.0021* | 0.0028* |
| CYP2D6 | rs28371725 | *41 | 22.10:g.42523805C>T | 0.0997 | 0.1231 | 0.0735 | 0.0727 | 0.0751* |
| CYP3A5 | rs776746 | *3 | 07.13:g.99270539C>T | 0.1183 | 0.0699 | 0.0739* | 0.0721* | 0.0775* |
| CYP3A5 | rs10264272 | *6 | 07.13:g.99262835C>T | 0.0073 | 0.0009 | 0.0022* | 0.0021* | 0.0025* |
| DPYD | rs3918290 | *2A | 01.10:g.97915614C>T | 0.0045 | 0.0048 | 0.0065 | 0.0070 | 0.0061 |
| DPYD | rs67376798 | (c.2846A>T) | 01.10:g.97547947T>A | 0.0039 | 0.0042 | 0.0035* | 0.0035 | 0.0036* |
| FVL | rs6025 | - | 01.10:g.169519049T>C | 0.0195 | 0.0242 | 0.0342 | 0.0344 | 0.0340 |
| TPMT | rs1800462 | *2 | 06.11:g.18143955C>G | 0.0023 | 0.0023 | 0.0029 | 0.0029 | 0.0031 |
| TPMT | rs1142345 | *3A, *3C | 06.11:g.18130918T>C | 0.0408 | 0.0404 | 0.0477 | 0.0477 | 0.0477 |
| UGT1A1 | rs4148323 | *6 | 02.11:g.234669144G>A | 0.0034 | 0.0014 | 0.0030* | 0.0028* | 0.0033* |
| VKORC1 | rs9934438 | (c.1173C>T) | 16.9:g.31104878G>A | 0.3786 | 0.3919 | 0.3914 | 0.3904 | 0.3934 |

*^*^ HWE deviations (p-values not shown).
Abbreviations: MAFs: minor allele frequencies; NCBI: National Center for Biotechnology Information; SMD: severe mental disorders; DPYD: dihydropyrimidine dehydrogenase; FVL: factor V Leiden; TPMT: thiopurine methyltransferase; UGT1A1: UDP-glucuronosyltransferase 1A1; VKORC1: vitamin K epoxide reductase complex 1; HWE: Hardy-Weinberg Equilibrium.*

Supplement table 4: Number of actionable PGx variants per individual in the sample

The number of actionable PGx variants (mutant alleles of genetic variants) per individual is presented based on the 19 identified clinically relevant PGx variants.

| **Number of actionable PGx variants** | **Total sample**  **(N=77 684)** | | **SMD case cohort**  **(N=51 464)** | | **Population cohort**  **(N=26 220)** | |
| --- | --- | --- | --- | --- | --- | --- |
|  | **N** | **(%)** | **N** | **(%)** | **N** | **(%)** |
| 0 | 14 | (0.02) | 7 | (0.01) | 7 | (0.03) |
| 1 | 1 584 | (2.0) | 1 064 | (2.1) | 520 | (2.0) |
| 2 | 8 419 | (10.8) | 5 622 | (10.9) | 2 797 | (10.7) |
| 3 | 17 511 | (22.5) | 11 564 | (22.5) | 5 947 | (22.7) |
| 4 | 20 163 | (26.0) | 13 312 | (25.9) | 6 851 | (26.1) |
| 5 | 16 577 | (21.3) | 10 975 | (21.3) | 5 602 | (21.4) |
| 6 | 9 634 | (12.4) | 6 434 | (12.5) | 3 200 | (12.2) |
| 7 | 3 189 | (4.1) | 2 117 | (4.1) | 1 072 | (4.1) |
| 8 | 534 | (0.7) | 333 | (0.6) | 201 | (0.8) |
| 9-11^1^ | 59 | (0.08) | 36 | (0.07) | 23 | (0.09) |

*^1^ Numbers in this row were grouped to prevent showing data <5.
Abbreviations: PGx: pharmacogenetics; SMD: severe mental disorders.*

Supplement table 5: Number of individuals with specific combinations of phenotypes

We compared combinations of CYP2C19-CYP2D6 phenotypes and VKORC1-CYP2C9 phenotypes.

| **Total sample (N=77 684)** | **CYP2D6_EM** | | **CYP2D6_IM** | | **CYP2D6_PM** | |
| --- | --- | --- | --- | --- | --- | --- |
|  | **N** | **(%)** | **N** | **(%)** | **N** | **(%)** |
| CYP2C19_UM | 1 821 | (2.3) | 990 | (1.3) | 132 | (0.2) |
| CYP2C19_RM | 12 392 | (16.0) | 6 791 | (8.7) | 865 | (1.1) |
| CYP2C19_EM | 21 235 | (27.3) | 11 242 | (14.5) | 1 379 | (1.8) |
| CYP2C19_IM | 11 916 | (15.3) | 6 393 | (8.2) | 809 | (1.0) |
| CYP2C19_PM | 1 091 | (1.4) | 564 | (0.7) | 64 | (0.1) |
|  | | | | | | |
| **SMD case cohort (N=51 464)** | **CYP2D6_EM** | | **CYP2D6_IM** | | **CYP2D6_PM** | |
|  | **N** | **(%)** | **N** | **(%)** | **N** | **(%)** |
| CYP2C19_UM | 1 185 | (2.3) | 651 | (1.3) | 78 | (0.2) |
| CYP2C19_RM | 8 159 | (15.9) | 4 573 | (8.9) | 547 | (1.1) |
| CYP2C19_EM | 14 078 | (27.4) | 7 390 | (14.4) | 918 | (1.8) |
| CYP2C19_IM | 7 968 | (15.5) | 4 243 | (8.2) | 550 | (1.1) |
| CYP2C19_PM | 707 | (1.4) | 375 | (0.7) | 42 | (0.1) |
|  | | | | | | |
| **Population cohort (N=26 220)** | **CYP2D6_EM** | | **CYP2D6_IM** | | **CYP2D6_PM** | |
|  | **N** | **(%)** | **N** | **(%)** | **N** | **(%)** |
| CYP2C19_UM | 636 | (2.4) | 339 | (1.3) | 54 | (0.2) |
| CYP2C19_RM | 4 233 | (16.1) | 2 218 | (8.5) | 318 | (1.2) |
| CYP2C19_EM | 7 157 | (27.3) | 3 852 | (14.7) | 461 | (1.8) |
| CYP2C19_IM | 3 948 | (15.1) | 2 150 | (8.2) | 259 | (1.0) |
| CYP2C19_PM | 384 | (1.5) | 189 | (0.7) | 22 | (0.1) |
|  | | | | | | |
| **Total sample (N=77 684)** | **CYP2C9_EM** | | **CYP2C9_IM** | | **CYP2C9_PM** | |
|  | **N** | **(%)** | **N** | **(%)** | **N** | **(%)** |
| VKORC1_WT | 19 147 | (24.6) | 8 700 | (11.2) | 1 014 | (1.3) |
| VKORC1_HET | 24 467 | (31.5) | 11 079 | (14.3) | 1 285 | (1.7) |
| VKORC1_HOM | 7 998 | (10.3) | 3 568 | (4.6) | 426 | (0.5) |
|  | | | | | | |
| **SMD case cohort (N=51 464)** | **CYP2C9_EM** | | **CYP2C9_IM** | | **CYP2C9_PM** | |
|  | **N** | **(%)** | **N** | **(%)** | **N** | **(%)** |
| VKORC1_WT | 12 716 | (24.7) | 5 764 | (11.2) | 686 | (1.3) |
| VKORC1_HET | 16 122 | (31.3) | 7 442 | (14.5) | 848 | (1.6) |
| VKORC1_HOM | 5 256 | (10.2) | 2 336 | (4.5) | 294 | (0.6) |
|  | | | | | | |
| **Population cohort (N=26 220)** | **CYP2C9_EM** | | **CYP2C9_IM** | | **CYP2C9_PM** | |
|  | **N** | **(%)** | **N** | **(%)** | **N** | **(%)** |
| VKORC1_WT | 6 431 | (24.5) | 2 936 | (11.2) | 328 | (1.3) |
| VKORC1_HET | 8 345 | (31.8) | 3 637 | (13.9) | 437 | (1.7) |
| VKORC1_HOM | 2 742 | (10.5) | 1 232 | (4.7) | 132 | (0.5) |

*Abbreviations: SMD: severe mental disorders; EM: extensive metabolizer; IM: intermediate metabolizer; PM: poor metabolizer; UM: ultrarapid metabolizer; RM: rapid metabolizer; VKORC1: vitamin K epoxide reductase complex 1; WT: wild-type carrier; HET: heterozygous carrier; HOM: homozygous carrier.*

References

1. van der Wouden CH, et al. Development of the PGx-Passport: A Panel of Actionable Germline Genetic Variants for Pre-Emptive Pharmacogenetic Testing. Clin Pharmacol Ther. 2019 Oct;106(4):866–73.

2. Pharmacogene Variation Consortium (PharmVar) [Internet]. [cited 2020 Feb 12]. Available from: www.PharmVar.org
